# Spread of *Coxiella burnetii* between dairy cattle herds in an enzootic region: modelling contributions of airborne transmission and trade

**DOI:** 10.1186/s13567-016-0330-4

**Published:** 2016-04-05

**Authors:** Pranav Pandit, Thierry Hoch, Pauline Ezanno, François Beaudeau, Elisabeta Vergu

**Affiliations:** INRA, LUNAM Université, Oniris, UMR1300 BioEpAR, CS40706, 44307 Nantes, France; MaIAGE, INRA, Université Paris-Saclay, 78350 Jouy-en-Josas, France

## Abstract

Q fever, a worldwide zoonotic disease caused by *Coxiella burnetii*, is a looming concern for livestock and public health. Epidemiological features of inter-herd transmission of *C. burnetii* in cattle herds by wind and trade of cows are poorly understood. We present a novel dynamic spatial model describing the inter-herd regional spread of *C. burnetii* in dairy cattle herds, quantifying the ability of airborne transmission and animal trade in *C. burnetii* propagation in an enzootic region. Among all the new herd infections, 92% were attributed to airborne transmission and the rest to cattle trade. Infections acquired following airborne transmission were shown to cause relatively small and ephemeral intra-herd outbreaks. On the contrary, disease-free herds purchasing an infectious cow experienced significantly higher intra-herd prevalence. The results also indicated that, for short duration, both transmission routes were independent from each other without any synergistic effect. The model outputs applied to the Finistère department in western France showed satisfactory sensitivity (0.71) and specificity (0.80) in predicting herd infection statuses at the end of one year in a neighbourhood of 3 km around expected incident herds, when compared with data. The model developed here thus provides important insights into the spread of *C. burnetii* between dairy cattle herds and paves the way for implementation and assessment of control strategies.

**Electronic supplementary material:**

The online version of this article (doi:10.1186/s13567-016-0330-4) contains supplementary material, which is available to authorized users.

## Introduction

Changes in social-economical, environmental and ecological factors are driving forces for the emergence of zoonotic infections [1]. In Europe, Q fever, a re-emerging zoonosis caused by the bacterium *Coxiella burnetii*, has seen a sharp rise in recent years, especially in the Netherlands with a large number of human cases which were attributed to livestock [2–4]. *C. burnetii* infections are common and subclinical in cattle, and generally result in reduced reproductive performance and abortions in primiparous cows [5, 6]. Infection in cattle herds is known to be widespread and enzootic [7]. Even though most of the recent human outbreaks are known to originate from small ruminants, intensive cattle farming with high prevalence could become a concern for public health [7]. Hence, investigation of infection dynamics in cattle herds at the first sign of its emergence is essential in the emergence-to-control continuum [8].

Cows acquire the infection through inhalation of *C. burnetii*. Various quantities of bacteria are shed by infectious animals through different routes [9]. Intra-herd infection dynamics of a dairy herd is mainly influenced by the heterogeneity of the shedding routes [10]. One of the important uncertainties concerning dynamics of infection lies in the contributions of the different routes in transmitting *C. burnetii* between livestock herds. Although airborne transmission of *C. burnetii* is a well-documented phenomenon [11–17], its precise contribution to the regional spread of *C. burnetii* between dairy cattle herds is poorly understood.

The same uncertainty holds for cattle trade. That is, no formal testing is conducted for the determination of the infection status of cows before sale or purchase. Recent studies have identified a risk of infection of dairy herds through airborne dispersion of bacteria and cattle trade [18, 19], but their respective quantitative contributions to the transmission are still unknown.

Here we present a novel individual-based metapopulation model in a stochastic framework including both spatial and temporal spread of *C. burnetii* between dairy cattle herds. Infection status and trade movement of animals between herds were individually tracked through time. The model has been applied to the case study of the Finistère department located in north-western France, characterised by a high density of dairy cattle, a windy marine west coast climate and a relatively flat terrain. The accuracy of model predictions was assessed based on meteorological, cattle trade and epidemiological data. Subsequently, the contributions of transmission routes to the regional spread of *C. burnetii* and their impact on the intra-herd infection dynamics were evaluated through the analysis of simulated scenarios.

## Materials and methods

### Summary of study approach and modelling framework

**Figure 1:**
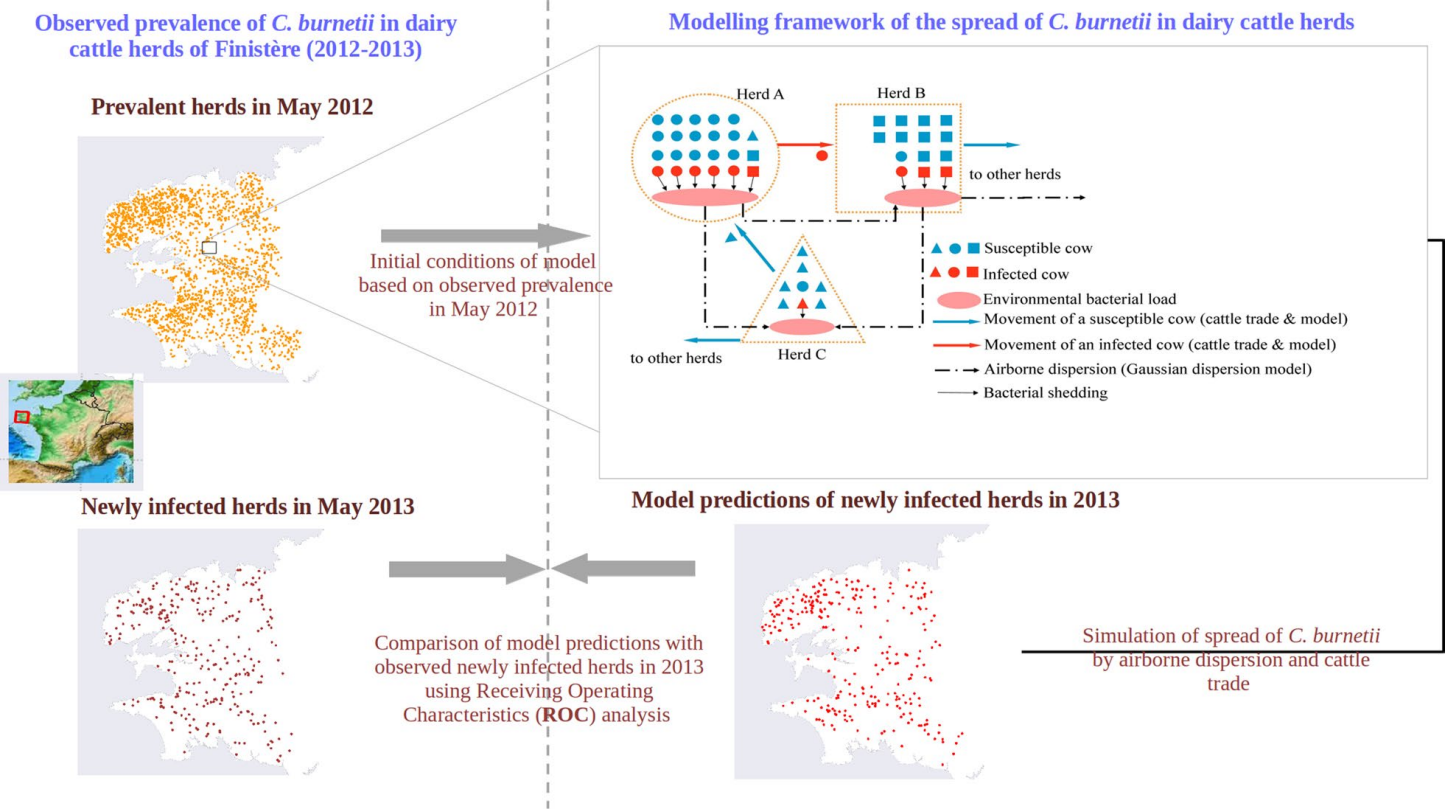
**Study approach and model framework.** Left panels represent observed data and right panels make reference to models. The modelling framework, describing intra-herd and inter-herd *C. burnetii* dynamics and the different transmission routes, is sketched in the top right panel: herd A is an initially prevalent herd, herd B is an initially susceptible herd further infected by cattle trade, and herd C is an initially susceptible herd further infected by airborne transmission. Inter-herd transmission is due to airborne dispersion (dashed arrows) and trade of cows (solid arrows). Intra-herd infection dynamics (detailed in Figure 2) is influenced by the bacteria shed by infected cows (red) contributing to infect susceptible cows (blue). Shapes representing cows correspond to the shape of their original herd. The initial conditions of the model are based on the observed regional prevalence in the Finistère department in 2012 (top left panel; the inset locates the Finistère department in France). Model predictions of newly infected herds in Finistère department for 2013 (bottom right panel) are compared with the observed newly infected herds in 2013 (bottom left panel) using receiver operating characteristics (ROC) analysis.

The different steps of the study approach and the modelling framework are summarised in Figure 1. The spread of *C. burnetii* between the dairy cattle herds of the Finistère department between May 2012 and May 2013 (data described in “Data” sub-section) was simulated using a metapopulation model (“Metapopulation model for regional spread of *C. burnetii*” sub-section). The initial conditions for simulations were generated according to the observed seroprevalence in tested herds in 2012.

The metapopulation model has three distinct components. The first one describes the spread of *C. burnetii* within each dairy cattle herd (intra-herd scale) along with their demographics (“Intra-herd dynamics of *C. burnetii*” sub-section). The second one describes *C. burnetii* transmission between the dairy cattle herds of the metapopulation due to cattle trade (“Inter-herd transmission due to cattle trade” sub-section). The third one describes *C. burnetii* between-herd transmission due to airborne dispersion (“Inter-herd infection dynamics due to airborne dispersion” sub-section).

Trade was incorporated into the model based on available data (details on data in “Data” sub-section). Airborne dispersion of *C. burnetii* was modelled using a Gaussian dispersion model (“Inter-herd infection dynamics due to airborne dispersion” sub-section) incorporating meteorological data (described in “Data” sub-section). The assessment of the accuracy of model predictions was performed by comparing model outputs and epidemiological data in 2013, using receiver operating characteristic analysis (“Assessment of model predictions” sub-section). Finally, the assessment of the relative contribution of cattle trade and airborne dispersion as transmission pathways between herds was performed through simulations. This final stage (not represented in Figure 1) is described in the “Assessment of the relative impact of transmission routes on the intra-herd and regional spread of *C. burnetii*” sub-section.

### Data

*Coxiella burnetii* infections are known to be enzootic in the cattle population of the Finistère department (north-western France). In May 2012, 2799 dairy cattle herds (69% of all the cattle herds in Finistère), were tested for antibodies against *C. burnetii* in bulk tank milk (BTM) using LSI Q fever enzyme—linked immunosorbent assay (ELISA) kit^®^ (LSI; Lissieu, France). Herds detected as seronegative in 2012 were retested again in May 2013. Data on geographical coordinates and number of cows in herds were also collected.

Furthermore, data on the movements of individual cows between dairy cattle herds and through markets of the Finistère department were extracted for the time period of May 2012–2013 from the national register (source: Groupements de Défense Sanitaire de Bretagne, France). In total, 835 out of 2799 dairy herds were involved (as a source or destination) in 2234 movements of cows (i.e. one movement corresponds to one cow being sold and purchased). In 182 herds, at least one animal during the study period was purchased and was not infected in May 2012. The mean number of animals sold per herd was equal to 4.7 (*n* = 474), the mean number of purchased animals was 5.3 (*n* = 491), the mean number of partners for selling was 1.4 and for buying animals was 1.6.

Wind speed data required for dispersion modelling were procured from the open access database of the European Centre for Medium Range Weather Forecasts [20]. Data on northward and eastward wind components for the Finistère department for the period of May 2012–2013 were extracted. Daily data were converted to weekly averages. Details of incorporation of the data into the model are given in Additional file 1.

### Metapopulation model for regional spread of *C. burnetii*

The metapopulation model, describing within-herd infection and demographic dynamics and between-herd transmission of *C. burnetii,* is made of three sub-models: an intra-herd dynamics model (“Intra-herd dynamics of *C. burnetii*” sub-section), an inter-herd model incorporating cattle trade (“Inter-herd transmission due to cattle trade” sub-section), and an inter-herd model incorporating airborne dispersion (“Inter-herd infection dynamics due to airborne dispersion” sub-section). The metapopulation model is a stochastic model in discrete time with one-week simulation time steps. Individual tracking of changes in infectious status and displacement between herds was performed for each animal in the metapopulation. It was restricted only to cows, since a previous study showed that nulliparous heifers and female calves were not observed to be infected [21]. Bulls and male calves were also excluded.

### Intra-herd dynamics of *C. burnetii*

The intra-herd dynamic model is an adapted version of the model of Courcoul et al. [10], whose transition parameters between health statuses were estimated from a longitudinal observational study using Bayesian estimation methods [22, 23]. It tracks individual health statuses of cows and accounts for the heterogeneity in shedding routes and levels of bacterial shedding. Moreover, the population dynamics of the herd was also incorporated through probabilities for culling events and the explicit representation of cow lactation cycles.

**Figure 2:**
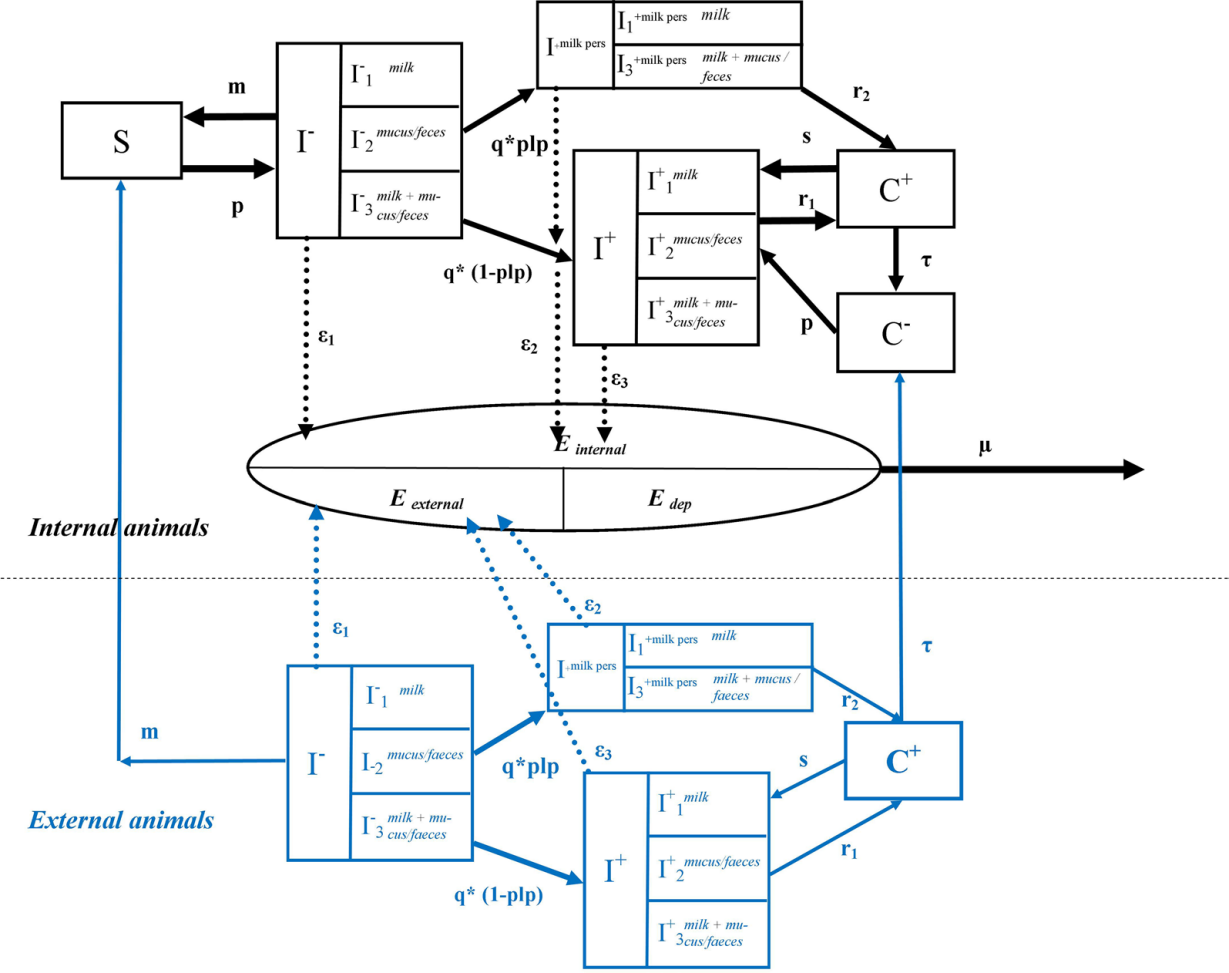
**Flow diagram describing the intra-herd spread of** ***C. burnetii*** **in a dairy cattle herd.** The diagram describes the health statuses of cows and transitions between these statuses, and environmental bacterial load of the herd (adapted from [10]). The blue section represents the infection dynamics of external animals, while the black section represents internal animals. *S*: susceptible, non-shedder cows without antibodies, *I* ^−^: shedder cows without antibodies, *I* ^+^: shedder cows with antibodies, *I* ^*milk pers*^: shedder cows with antibodies shedding in milk in a persistent way, *C* ^+^: non-shedder cows with antibodies, and *C* ^−^: non-shedder cows without antibodies which were infected and had antibodies in the past. *I* ^−^ and *I* ^+^ cows are in the shedding route category 1 if they shed in milk only, 2 if they shed in vaginal mucus/faeces only, and 3 if they shed in milk and vaginal mucus/faeces. *I* ^*milk pers*^ cows are in the shedding category 1 if they shed in milk only, and 3 if they shed in milk and vaginal mucus/faeces. *E* represents the force of infection related to the bacterial contamination of the environment. *E*_*local*_ corresponds to the part of the force of infection due to internal animals, whereas *E*_external_ is due to external animals and *E*_*dep*_ is due to bacteria deposited from other infectious herds by airborne transmission. ε1, ε2 and ε3 are the quantities of contributions to *E* during a time step by cows in statuses *I* ^−^, *I* ^+^, and *I* ^*milk pers.*^, respectively. These quantities are the sum of all quantities of bacterial load shed by all the shedders through all the shedding routes and reaching the environment of the herd. Details of the shedding levels and the proportions of cows shedding through different routes are given in Table 2. Description and values of the parameters used are given in Table 1.

After infection, cows of the herd undergo transitions between health statuses (Figure 2, with parameters defined in Table 1) [10]. These health statuses were defined according to the seropositivity and shedding characteristics: susceptible, non-shedder, sero-negative cows (*S*); infectious shedder, sero-negative cows (*I*^−^); shedder, with antibodies (*I*^+^) and permanently shedding in milk at higher levels (*I*^+milk pers^); carriers non-shedders, with antibodies (*C*^+^); and previously infected cows, without antibodies (*C*^−^). Shedding cows could shed the bacteria through milk, mucus/faeces, or through both routes at low, medium, or high levels of shedding (*Qty*), with probability distributions of shedding levels depending on the infection status. The quantities of the bacteria reaching the environment, shed by all the shedders in statuses *I*^−^, *I*^+^, and (*I*^+milk pers^), respectively, through all the shedding routes were denoted by ε_1_, ε_2_ and ε_3_. All the parameters related to the heterogeneity of shedding are presented in Table 2 [10].

**Table 1:** Parameters of the intra-herd infection dynamics for a dairy cattle herd (adapted from [23]).

Parameter	Definition	Value
*m*	Transition probability *I* ^−^ ⇒ *S*	0.7
*q*	Transition probability *I* ^−^ ⇒ *I* ^+^	0.02
*pIp*	Proportion of cows going from *I* ^−^ to *I* ^+^ and becoming *I* ^*milk pers*^	0.5
*r1*	Transition probability *I* ^−^ ⇒ *C* ^+^	0.2
*r2*	Transition probability *I* ^*milk pers*^ ⇒ *C* ^+^	0.02
*s*	Transition probability *C* ^+^ ⇒ *I* ^+^	0.15
*τ*	Transition probability *C* ^+^ ⇒ *C* ^−^	0.0096
*μ*	Proportion of bacteria eliminated due to death and plume generation (can be written as $\mu_{death} + \mu_{plume source}$)	0.2
*p*	Infection probability	1−*e* ^−*E*^
*ρ* ^*m*/*f*^	Proportion of bacteria shed through mucus/faeces filling the environment compartment	0.28
*ρ* ^*m*^	Proportion of bacteria shed through milk filling the environment compartment	0.125*ρ* ^*m*/*f*^

**Table 2:** Description and probability distributions used for the different shedding routes and levels (from [10]).

	Parameter	Definition	Value
*α*	*α*_1_, milk	Probability distribution of the shedding routes for the *I* ^−^ cows	0.31
	*α*_2_, mucus/faeces		0.62
	*α*_3_, milk + mucus/faeces		0.07
*β*	*β*_1_, milk	Probability distribution of the shedding routes for the *I* ^+^ cows after 4 weeks post-calvingss	0.61
	*β*_2_, mucus/faeces		0.33
	*β*_3_, milk + mucus/faeces		0.06
*β*_*calv*_	*β*_*calv1*_, milk	Probability distribution of the shedding routes for the *I* ^+^ cows in the 4 first weeks post-calving	0.14
	*β*_*calv3*_, mucus/faeces		0.5
	*β*_*calv3*_, milk + mucus/faeces		0.36
*γ*	*γ*_1_, milk	Probability distribution of the shedding routes for the $I^{milk pers}$ cows after 4 weeks post-calving	0.83
	*γ*_3_, milk + mucus/faeces		0.17
*γ*_*calv*_	*γ*_*calv1*_, milk	Probability distribution of the shedding routes for the $I^{milk pers}$ cows in the 4 first weeks post-calving	0.25
	*γ*_*calv3*_, milk + mucus/faeces		0.75
Q1	Low level	Probability distribution of the shedding levels for all the *I* ^−^ and for the *I* ^+^ shedding in mucus/faeces after 4 weeks post-calving	0.85
	Mid-level		0.15
	High level		0
Q2	Low level	Probability distribution of the shedding levels for the *I* ^+^ shedding in milk after 4 weeks post-calving	0.4
	Mid-level		0.5
	High level		0.1
Q3	Low level	Probability distribution of the shedding levels for all the *I* ^+^ in the 4 first weeks post-calving	0.25
	Mid-level		0.25
	High level		0.5
Q4	Low level	Probability distribution of the shedding levels for the $I^{milk pers}$ shedding in mucus/faeces after 4 weeks post-calving	0.6
	Mid-level		0.4
	High level		0
Q5	Low level	Probability distribution of the shedding levels for all the $I^{milk pers}$ shedding in milk and for the $I^{milk pers}$ in the 4 first weeks post-calving	0.15
	Mid-level		0.6
	High level		0.25

The probability of transition from *S* to *I*^−^, i.e. the probability of a susceptible cow of herd *i* to acquire a *C. burnetii* infection at time *t* was defined as

1 $$p_{i} (t) = 1 - e^{{ - (E_{i} (t - 1))}} .$$

In Equation 1, *E*_*i*_*(t)* [number of bacteria/s] is the force of infection related to the bacterial contamination of the environment at time *t* and is equal to:

2 $$ E_{i} (t) = E_{i} (t -1)(1 -\mu ) + E_{i,internal} (t - 1) + E_{i,external} (t - 1) + \sum\nolimits_{j} {E_{i,j,dep} (t -1)}$$

Furthermore, *E*_*i*_*(t)* corresponds to the bacterial load at time *t* (expressed in infectious doses), times the contact rate between animals and the environment, times the probability that a contact of a susceptible animal with an environment contaminated by one infectious dose leads to a successful infection event. A similar formulation of the probability of infection has been proposed previously for aerosol infection of *C. burnetii* [24]. Equation 2, which is the main adaptation of the model of Courcoul et al. [10], allowed us to account for contributions to the force of infection from three different sources: bacteria shed by internal animals (*E*_*i*,*internal*_); bacteria shed by external animals, introduced into the herd by purchase (*E*_*i*,*external*_); and bacteria coming from the environment of all other neighbouring herds *j*, imported through airborne dispersion (*E*_*i*,*j*,*dep*_). Cows born in the same herd or susceptible (*S*) at the time of purchase were called internal animals. Cows which were infected outside the herd and that were shedders ($I^ - ,I^{ + } \;{\text{or}}\;I^{ + milk\;pers}$) or carriers (*C*^+^) at the time of purchase were called external animals. The infection dynamics of the internal and external animals were assumed to be identical.

The loss of bacteria from the environment (*μ*) encompasses natural death (*μ*_*death*_) and loss of bacteria that are transmitted airborne to the outdoor environment (*μ*_*plumesource*_):

3 $$\mu = \mu_{death} + \mu_{plume\;source}$$

The complete system of equations describing the infection dynamics at herd level is provided in Additional file 1.

#### Inter-herd transmission due to cattle trade

When an infectious cow moves from one herd to another, it contributes to the spread of *C. burnetii* within the destination herd (i.e. to the term *E*_*i*,*external*_ in Equation 2 for herd *i*). All movements of cows between dairy cattle herds in the Finistère department were modelled according to observed trade data on the source and destination herds, the movement date, and the age of moved cows. The only information not present in the data was the animal health status. Hence, while modelling cattle trade, for each individual movement observed between May 2012 and May 2013, an animal in the same lactation year (parity) as the one recorded in the dataset was randomly chosen from the source (selling) herd to move to the destination herd. The probability of trading an infectious cow therefore was related to the proportion of infectious animals in the given lactation year in the source herd. Because of the relatively low time spent by cows in markets (less than 24 h) during trading and the lack of information about such events, it was assumed that there was no transmission between cows following any possible interaction between them in markets. Due to the lack of information about the prevalence outside the concerned study region, only movements of cows between the herds within the study region were considered.

#### Inter-herd infection dynamics due to airborne dispersion

In infected herds, part of the environmental bacterial contamination represented by *E* compartment (*μ*_*plumesource*_), becomes airborne and thus is transmitted to the outdoor environment (Equation 3). The small cell variant (SCV) of *C. burnetii* is known to be resistant to various environmental conditions [25]. Plume transportation was modelled using a Gaussian dispersion equation which accounts for phenomena such as transportation, settling, and gravitation, presented in Ermak [26] and Stockie [27].

4 $$C_{{i,j,(x,y,z)}} = \frac{{Q_{j} }}{{2\pi U\sigma _{y} \sigma _{z} }}e^{{\left( {\frac{{ - y^{2} }}{{2\sigma _{y}^{2} }}} \right)}} e^{{\left( {\frac{{ - W_{{set}} (z - h)}}{{2K_{z} }} - \frac{{ - W_{{set}}^{2} \sigma _{z}^{2} }}{{8K_{z}^{2} }}} \right)}} \left[ {e^{{\frac{{ - (z - h)^{2} }}{{2\sigma _{z}^{2} }}}} + e^{{\frac{{ - (z + h)^{2} }}{{2\sigma _{z}^{2} }}}} - \frac{{\sqrt {2\pi } W_{0} \sigma _{z} }}{{K_{z} }}e^{{\left( {\frac{{W_{0} (z + h)}}{{K_{z} }} + \frac{{W_{0}^{2} \sigma _{z}^{2} }}{{2K_{z}^{2} }}} \right)}} \text{erfc}\left( {\frac{{W_{0} \sigma _{z} }}{{\sqrt {2K_{z} } }} + \frac{{(z + h)}}{{2\sigma _{z}^{2} }}} \right)} \right]$$

The concentration *C*_*i*,*j*,(*x*,*y*,*z*)_ is defined as the number of bacteria [m^−3^] reaching herd *i* from source herd *j* (where *x*, *y* are differences in respective coordinates of herds *i* and *j* on x-axis and y-axis, and *z* the height at which the plume is generated in source herd *j*).

Furthermore, the emission rate *Q*_*j*_ [number of bacteria/s] = E_j_*μ*_*plumesource*_ is the part of force of infection which becomes source for plume generation in source herd *j*; *U* [m/s] is the wind velocity; σ_y_ [m] and σ_z_ [m] are the standard deviations for dispersion coefficients, parameterised as $\sigma_{y} ({\text{x}}) = a_{y} x^{{b_{y} }}$ and $\sigma_{z} ({\text{x}}) = a_{z} x^{{b_{z} }}$ with *a*_*y*_*, a*_*z*_*, b*_*y*_*, b*_*z*_ corresponding to the atmospheric stability class C (3-5 m/s wind velocity, slightly unstable environment); *W*_*0*_ [m/s] = *W* − 0.5*W*_*set*_, where *W* [m/s] is the deposition velocity due to gravitation and *W*_set_[m/s] is the settling velocity, fixed to $\frac{{2\varphi gr^{2} }}{9\eta }$ with *φ* [kg/m^3^] the particle density, *r* [m] the particle radius, *η* [kg/m s] the dynamic viscosity of air, and *g* [m/s^2^] the gravitational acceleration; *h* [m] is the height of reception at destination herd; and *K*_*z*_ [m^2^/s] is the coefficient of eddy diffusivity set to $K_{z} = 0.5a_{z} b_{z} Ux^{{(b_{z} - 1)}}$. In the last term of Equation 4, *erfc* is the complementary error function (erfc (*x*) = 1 − erf(*x*)) resulting from the approximation of the solution of the partial differential equation of advection–diffusion. Parameters were taken from the standard model presented in Stockie [27]. Additional details on these dispersion related parameters are given in Table 3.

**Table 3:** Parameters of the airborne transmission.

Parameter	Definition	Estimation	Unit
*g*	Gravitational acceleration	9.8	m s^−2^
*z*	Height of plume generation	4	m
*h*	Height of plume reception	4	m
*η*	Dynamic viscosity of air	1.8 × 10^−5^	kg m^−1^ s^−1^
*φ*	Density of particles	1150 [42]	kg m^−3^
*r*	Radius of particle	10^−6 a^	m^a^
*W*	Deposition velocity	0.01 [27]	m s^−1^
*a*_*y,*_ *a*_*z*_	Guifford–Pasquill stability	0.34, 0.27 [27]	m^(1−b)^
*b*_*y,*_ *b*_*z*_	Class “C” stability parameters	0.82, 0.82 [27]	

^a^ Approximated from [43].

Bacteria arriving from a neighbouring herd *j* through airborne transmission accumulate in compartment *E*_*i,j,dep*_, given by *E*_*i,j,dep*_ = *area*_*i*_*W* *C*_*i,j,(x,y,z)*_, where the area for each herd (*area*_*i*_) was approximated using average space recommendation for a cow and the number of cows in a given herd. *W* and *C*_*i,j,(x,y,z)*_ were presented in Equation 4.

### Initial conditions and outputs

Initial conditions were simulated according to the distribution of intra-herd seroprevalence observed in May 2012 in the Finistère department (“Data” sub-section). For all the positive herds in May 2012, an intra-herd model was run, thereby neglecting between-herd contacts and starting from one initially infected cow. Each of these intra-herd models was run until the herd-specific simulated seroprevalence reached a value ranging in the interval of the expected mean ± 1 standard deviation of the herd-specific observed seroprevalence, as provided in Taurel et al. [28]. Once the initial conditions were obtained for each herd initially infected, the between-herd spread of *C. burnetii* by both trade and airborne dispersion was simulated over 1 year, to assess if the model can predict similar spread of *C. burnetii* as observed in May 2013.

The metapopulation model was used to predict the statuses in May 2013 of herds initially susceptible in May 2012 (where introduction of *C. burnetii* in herds was defined as the generation of the first infection among internal animals). Herds purchasing infectious animals previous to the generation of the first local infection were designated as being infected by cattle trade, the rest of the incident herds were attributed to airborne transmission. The probability of infection (PI) was also estimated for each incident herd, based on the proportion of runs it experienced with infection: PI = (number of runs with at least one local infection)/(total number of runs). Herds were predicted positive by the model if their predicted PI was higher than a threshold, which was calibrated according to the available data, as described in “Assessment of model predictions” sub-section. Moreover, for each incident herd, intra-herd seroprevalence, proportion of shedders (defined in Additional file 1, Eqs. 7 and 8), extinction rate (proportion of runs with no shedding and no seropositive cow at the end of the simulation among the runs where the herd was infected), and herd incubation period (time elapsed between exposure to the identified cause and generation of the first local infection) were also recorded as model outputs. Descriptive statistical measures (mean, median, standard deviation, and percentiles) of seroprevalence and proportion of shedders in incident herds were calculated over runs in which herds experienced an infection.

All the outputs of the study were generated after 100 iterations (which proved to suffice in terms of mean stabilisation) of the metapopulation model.

### Assessment of model predictions

The accuracy of the metapopulation model in predicting the infection status in May 2013 for all of the initially susceptible herds as observed in May 2012 was assessed by receiver operating characteristics (ROC) analysis. A ROC analysis consists in evaluating the performance of a classifier, in terms of Sensitivity (*Se*) and Specificity (*Sp*), in detecting binary behaviour (here infected/non-infected) for different discrimination thresholds. For each initially susceptible herd, the simulated PI (our classifier) provided a prediction of the herd infection status after comparison to a threshold. This predicted status was compared against the herd observed status in 2013 (herd level analysis).

To assess possible improvements in prediction, we relaxed the spatial precision in the ROC analysis and compared the output for a neighbourhood around an expected incident herd (neighbourhood level analysis). The comparison was done for neighbourhood distances of multiple radii (1, 2, 3 or 4 km). Specificity (*Sp*) for the neighbourhood level analysis was considered as equal to that of herd level analysis. AUC (Area Under the Curve) was used to assess the model performance.

The optimum cut-off (threshold) values for PI to classify herds as positive or negative were selected based on three criteria: (i) equality of sensitivity (*Se*) and specificity (*Sp*), *Se* = *Sp*; (ii) maximum accuracy *(Acc*_*max*_*)*, where *Acc* = (true positive herds + true negative herds)/(total population of herds), or, equivalently, *Acc* = *Se* × Prevalence + *Sp* × (1−Prevalence); and (iii) maximum Youden index (*J*_*max*_), where *J* = *Se* + *Sp* − 1 [29].

To identify regions with high risk of incidence, a spatial cluster analysis for predicted positive herds was done using a Poisson model (SatScan^®^) with a null hypothesis of expected number of cases in each area proportional to its population size, thus adjusting the model for cow density. Definition of a positive herd was based on the optimum PI cut-off suggested by the ROC analysis.

A preliminary sensitivity analysis was done to assess the robustness of the model predictions with respect to parameter variations. In a detailed sensitivity analysis conducted on the intra-herd model by Courcoul et al. [10], three significantly sensitive parameters were found: *Q1*, *ρ*, and *µ*. Along with these three parameters, we tested three additional parameters from the dispersion model (*κ*, *r*, *W*). The values chosen to be tested in the sensitivity analysis (parameter definition and values in Table 4) were those used in [10] for *Q1*, *ρ*, and *µ*. For *κ*, *r*, and *W*, the standard value was varied by fifty percent, in the limits of biological plausibility. Each parameter was varied independently of other parameters (univariate sensitivity analysis) and the effect of these variations was evaluated on three model outputs (the relative contribution of airborne transmission to new herd infections, the number of incident herds, and the proportion of shedders in incident herds).

**Table 4:** Parameters considered for the model sensitivity analysis.

Parameter	Definition	Standard value	Values tested in sensitivity analysis			
Q1	Probability distribution of the shedding levels of all the *I* ^−^ and for the *I* ^+^ shedding in mucus/faeces after 4 weeks post calving	Distribution I	Distribution II	Distribution III	Distribution IV	
Low-level		0.85	0.6	0.25	0.15	
Mid-level		0.15	0.4	0.25	0.6	
High-level		0.0	0	0.5	0.25	
ρ	Proportion of bacteria shed through mucus and faeces filling the compartment	0.28	0.05	0.15	0.35	0.5
µ	Elimination rate of *C. burnetii* from the herd environment	0.2	0.1	0.5	0.8	
κ	Ratio between *µ*_*plume source*._ and *µ*	0.5	0.25	0.75		
r	Radius of a fomite particle	1e−6	0.5e−6	1.5e−6		
W	Deposition velocity due to gravitation	0.01	0.005	0.015		

### Assessment of the relative impact of transmission routes on the intra-herd and regional spread of *C. burnetii*

We tested four scenarios to understand the role of cattle trade and airborne dispersion both independently and in association with one another: absence of inter-herd transmission (scenario A), transmission only by movement of animals (scenario B), airborne transmission only (scenario C), and both transmission routes (scenario D). Incidence dynamics at the herd level, the total number of incident herds, and the dynamics of shedder cows in incident herds were the outputs of interest. We further focused on scenario D and evaluated the relative roles of the two transmission pathways in introducing *C. burnetii* in herds. We used a Mann–Whitney U test to compare PI, extinction rate, and herd incubation period between herds infected solely by airborne transmission and herds infected solely by trade. A similar analysis was done on a subset of herds at risk of acquiring *C. burnetii* through both routes, i.e. those herds that purchased animals and were also exposed to *C. burnetii* due to airborne transmission.

## Results

### Incidence prediction and agreement with observed data

**Figure 3:**
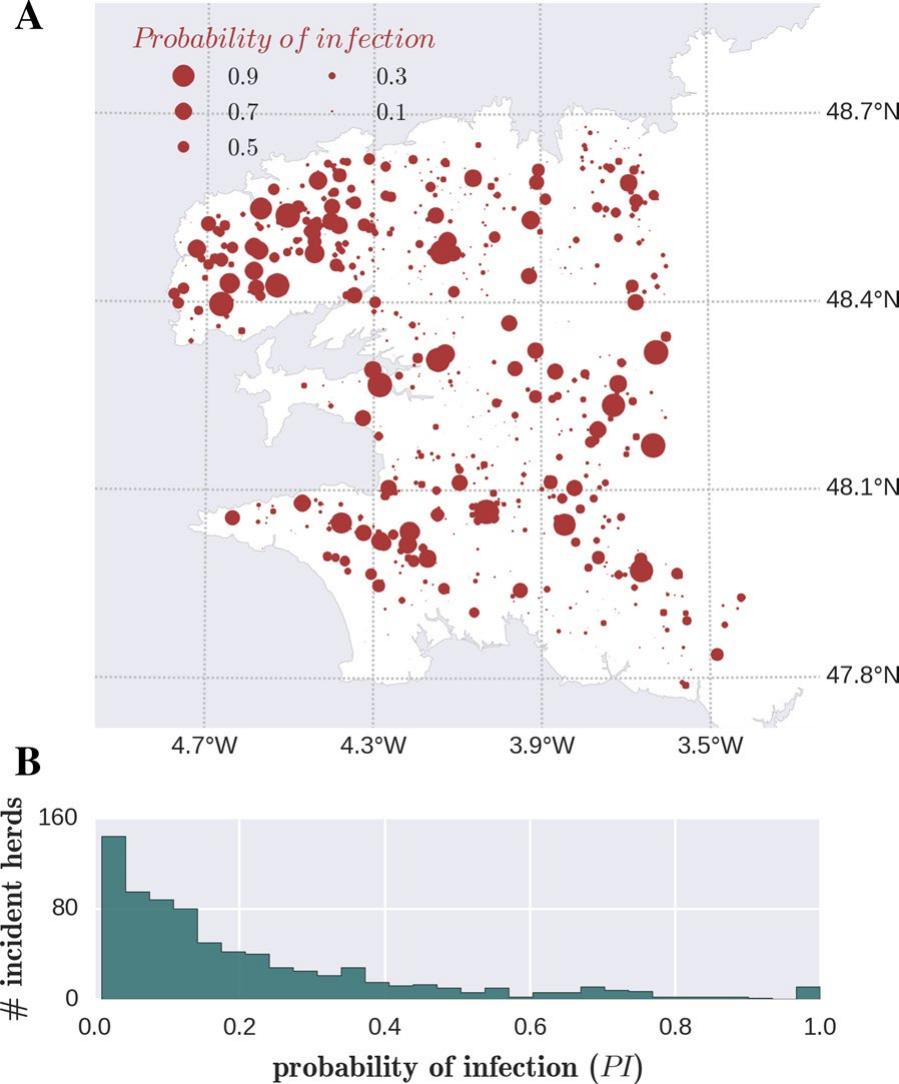
**Infection probabilities of initially susceptible herds.** Simulated probability of infection (PI) by *C. burnetii* after one year of inter-herd spread, for herds observed to be infection-free in May 2012. (**A**) Map of the Finistère department in north-western France with the locations of incident herds (bubbles sizes are proportional to PI). (**B**) PI distribution amongst the 768 herds that get infected at least once in the simulations.

According to the epidemiological data, in May 2012, 69.3% (*n* = 1941) of the herds were found seropositive (referred hereafter as prevalent herds). Among the 858 initially negative herds, 826 were retested one  year later in May 2013, and 306 were found positive (they represent the observed incident herds in the study). According to the model, out of 858 susceptible herds at the beginning of the simulation, 768 got infected at least once over the total number of runs. The PI predicted for incident herds showed spatial heterogeneity (Figure 3A). Most of incident herds had low values of PI. Out of 768 herds, 38.8% of herds had PI < 0.1, while only 1.5% of herds had PI ≥ 0.9 (Figure 3B).

**Figure 4:**
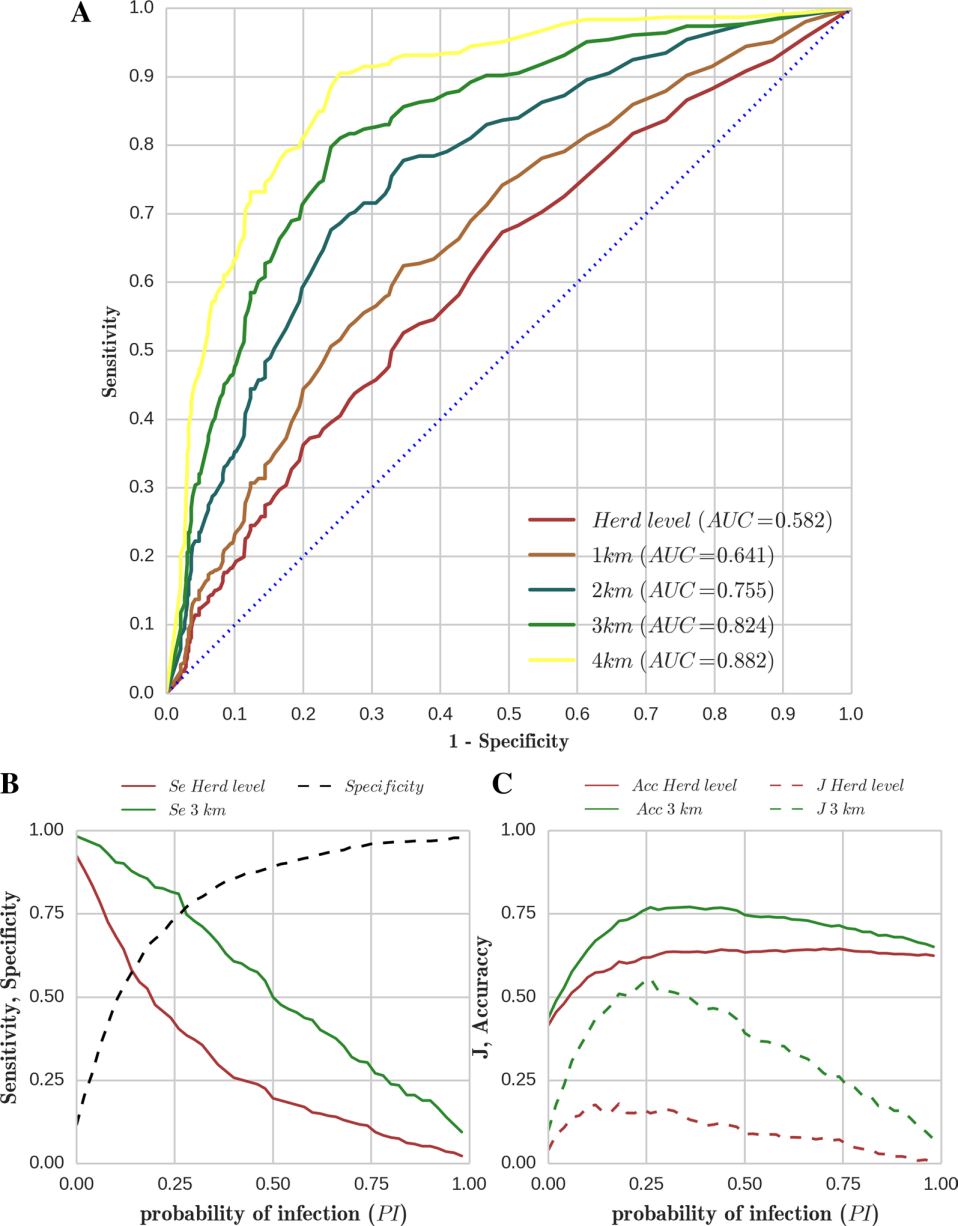
**Receiver operating characteristic (ROC) analysis of model predictions.** ROC analysis (data are the reference) for the simulated probability of infection (PI) by *C. burnetii* after 1 year of inter-herd spread, for herds initially susceptible. (**A**) ROC curves for herd level analysis and neighbourhoods of 1, 2, 3, 4 km. The area under the curve (AUC) for each analysis is indicated in the legend. (**B**, **C**) Variation of the four indicators [Sensitivity (*Se*), Specificity (*Sp*), Accuracy (*Acc*), Youden Index (*J*)] used for building the three criteria [*Se* = *Sp*, max(*Acc*), max(*J*)] to optimise the cut-off of PI for the classification of herds as positive and negative. Calculations were performed at herd level and for a neighbourhood of 3 km. Model *Sp* was assumed to be identical over the different neighbourhoods and thus is shown as a single line.

The model had moderate agreement with data at herd level. It performed better for predictions at the neighbourhood level (Figure 4A). In the radius of 2, 3, 4 km, there were on average respectively 1.7, 3.8, and 6.6 initially susceptible neighbour herds around an expected incident herd in the Finistère department. The gain in the model predictive ability in terms of AUC with the increase in the neighbourhood radius was weighed against the loss in the accuracy of model predictions arising because of an increase in the number of susceptible herds the calculations rely on. This resulted in a subjective compromise where a neighbourhood of 3 km was retained for further analyses of model results.

Optimum cut-off values for PI were estimated based on three criteria, as described in the “Assessment of model predictions” sub-section and illustrated in Figure 4B, C. At herd level, the model performed better at PI cut-off = 0.11 for the first and third criteria (*Se* = *Sp* = 0.58, *J*_*max*_ = 0.15), and at PI cut-off = 0.61 for the second one (*Se* = 0.1, *Sp* = 0.95, *Acc*_*max*_ = 0.64). For a radius of 3 km, the optimal cut-off was found to be 0.21 based on the first criterion (*Se* = *Sp* = 0.75) 0.25 based on the second one (*Se* = 0.71, *Sp* = 0.80, *Acc*_*max*_ = 0.76), and 0.15 according to the third one (*Se* = 0.86, *Sp* = 0.66). Details of the *Se*, *Sp*, *Acc*, *J*, predicted incidence, contribution of airborne transmission to the incidence, and the spatial distribution of incident herds at these cut-offs are given in Additional files 2 and 3.

**Figure 5:**
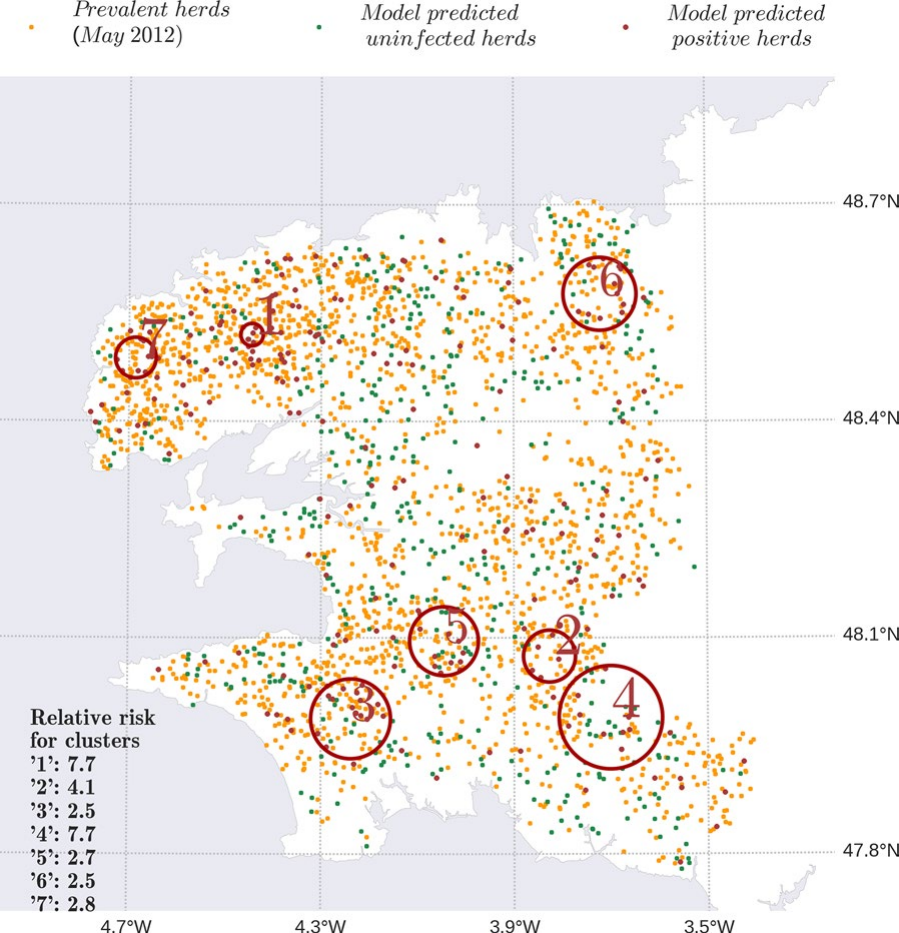
**Spatial clustering of infection probability in the Finistère department.** Seven statistically significant spatial clusters (circled in red) with high relative risk (values are indicated in the legend) of presence of predicted incident herds (red dots), initially susceptible and infected by *C. burnetii* after one year of inter-herd spread. The positivity of a herd was defined based on a cut-off value of 0.25 for the probability of infection. Herds initially seroprevalent according to the data (orange dots) and herds which remain uninfected (green dots) are also represented.

We performed the subsequent cluster analysis using a cut-off value of 0.25 (i.e. herds were declared as positive if PI ≥ 0.25), which provided the best results with respect to the three criteria at the selected radius. According to the cluster analysis, herds predicted as positive by the model at the cut-off of 0.25 had seven non-overlapping statistical clusters: three in the north and four in the south of the Finistère department (Figure 5). A small cluster (cluster 1, Figure 5) in northern Finistère department had the highest relative risk of 7.7, when compared with herds outside the cluster.

**Figure 6:**
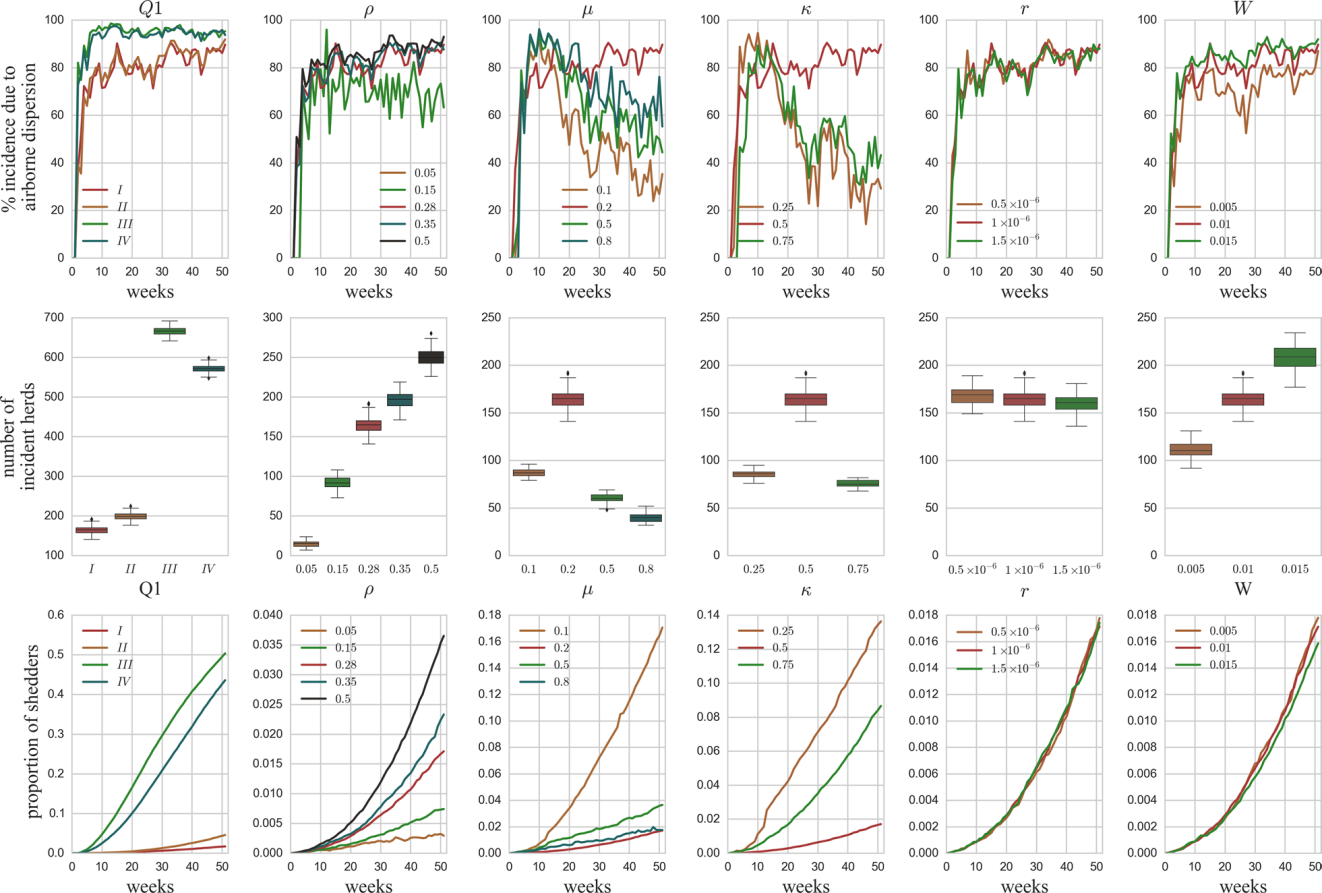
**Univariate sensitivity analysis.** Sensitivity analysis of three dynamical outputs of the model (mean proportion of herd incidence due to airborne transmission (top line), number of incident herds (middle line), and mean proportion of shedders in incident herds (bottom line) over 100 stochastic iterations of the model) with respect to the variation in six parameters (from the left to the right, each column corresponds to a single parameter: *Q1, ρ, µ*, *κ*, *r*, and *W*) (details in Table 4).

Model outputs were sensitive to *Q1*, *ρ*, *µ*, and *κ*, whereas very little perturbations were induced by variations of particle size, *r*, and deposition velocity, *W* (Figure 6). The results of the sensitivity analysis suggested that, despite a considerable sensitivity of the model to the parameters tested (except for *r*), the relative contribution of airborne transmission in the simulated incidence remained higher than the contribution of cattle trade, regardless of the parameter values tested, except for some values of *κ* and *µ* for which this trend was reversed in the last 6 months of the simulation duration.

### Contribution of transmission pathways to the regional spread

Airborne transmission was responsible for the infection of the majority of incident herds as predicted by the model at all the optimum PI cut-offs derived in the ROC analysis. The contribution to the total number of infections as a function of these cut-offs varied from 57 to 86% at the herd level, and from 75 to 83% with a radius of 3 km (see Additional file 2). The sensitivity analyses showed that airborne transmission contributed to more than 50 and 70% of the new herd infections in 88 and 63% of the tested situations, respectively (Figure 6).

**Figure 7:**
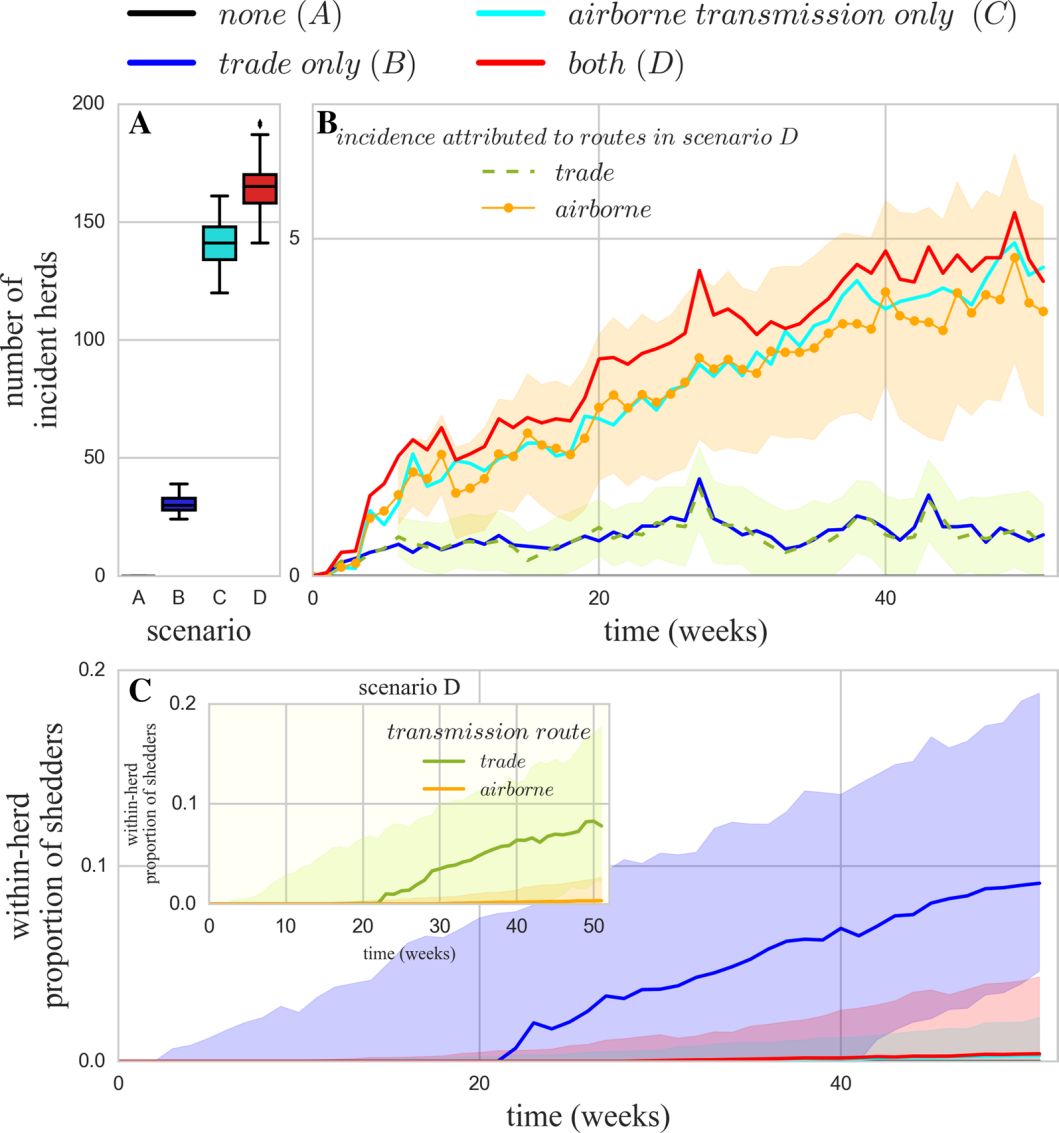
**Infection dynamics of** ***C. burnetii*** **spread over one** **year in four simulated scenarios.** Absence of inter-herd transmission (**A**, black, not visible as all the results are null), transmission by cattle trade only (**B**, blue), transmission by airborne dispersion (**C**, cyan), and presence of both transmission routes (**D**, red). The subdivision of scenario D based on the identified cause of herd infection is also represented (due to animal trade—orange; by airborne dispersion—green). (**A**) Distribution of the total number of predicted incident herds. (**B**) Dynamics of incidence (mean over 100 runs). Shaded regions for the subdivisions of scenario D represent 95% empirical confidence intervals. (**C**) Median proportion of intra-herd shedders and 80^th^ percentile (represented by shaded area) for all the scenarios. Inset figure shows the proportion of shedders (median and 80th percentile) for subdivisions of scenario D. Median and percentiles are calculated for runs where herds experienced infection (sample sizes are 16 733 for D, 13 814 for C and 3617 for B).

Figure 7 illustrates the effect of airborne transmission and cow trade on the regional spread of *C. burnetii*. More incident herds were seen in scenarios comprising airborne transmission (C and D, at least five times more incident herds on average than in scenario B; Figure 7A, B). Further analysis of scenario D carried out in the second approach provided similar results for the predicted incidence. In all of the 100 iterations of the standard stochastic model, 92% of all the new herd infections were attributed to airborne transmission, while the rest (8%) was attributed to cattle trade. The incidence dynamics over the time period attributed to these two transmission routes when acting simultaneously exhibit close coherence with the incidence predicted in scenarios B and C, where each transmission route was considered separately (Figure 7B). Incidence attributed to airborne transmission (scenario C and deconvolution of scenario D) shows an initial rapid increase followed by a steady growth, while the incidence attributed to cattle trade was comparatively low and constant throughout the simulation period (scenario B and deconvolution of scenario D). The analysis performed on the subset of herds at risk from getting infected through both routes, with parameter values corresponding to the standard scenario, led to results consistent to those obtained for the whole population of initially susceptible herds. On average, the majority of the new herd infections were due to airborne transmission (65%).

### Impact of transmission pathways on the intra-herd dynamics

**Figure 8:**
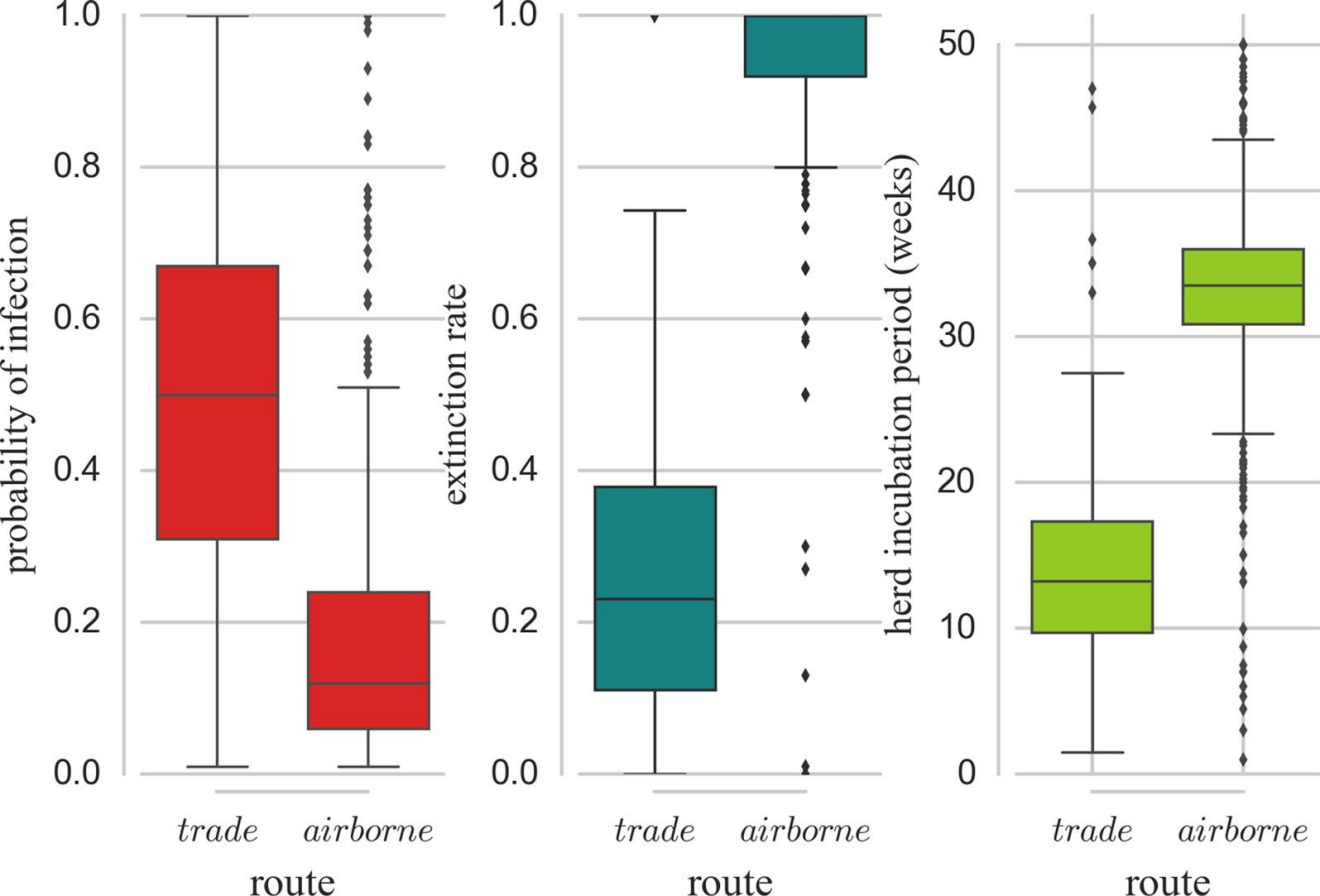
**Distribution of probability of infection (PI), extinction rate, and herd incubation period.** Distribution of simulated PI, extinction rate, and time before generation of the first infection after exposure to the cause of infection in *C. burnetii* newly infected herds (one year of simulated infection dynamics) by cattle trade and airborne transmission. Each box contains values between the first and the third quartiles. Horizontal lines outside boxes correspond to the first quartile −1.5× interquartile range and the third quartile +1.5× interquartile range. Horizontal lines within the boxes correspond to medians.

The impact of presence and absence of a transmission route on the intra-herd infection dynamics was highlighted in the four scenarios. The scenario involving only trade (B) shows higher proportion of shedders (Figure 7C) and intra-herd seroprevalence (not shown) in incident herds, than scenarios involving airborne transmission only (C) or both transmission pathways (D). When both transmission routes were accounted for, herds infected due to airborne transmission showed significantly lower levels of shedding animals than those infected after purchasing an infectious cow (Figure 7C inset). Other representative parameters of the infection dynamics were also found statistically significantly different (*p* < 0.05, Figure 8). PI was higher in herds infected by cattle trade, while the extinction rate was higher in airborne infected herds. These latter also took significantly longer time to generate the first local infection after exposure to the respective cause than herds infected by cattle trade.

Variation in the intra-herd dynamics (proportion of shedders) followed similar trends when performed on the subset of herds exposed to both transmission routes as seen in the analysis done on all susceptible herds. Also, for all outputs considered (PI, time after infection and extinction rate), statistically significant difference in herds infected by airborne transmission and herds infected by cattle trade was found.

## Discussion

Our findings showed that airborne transmission and movement of cows both affected the regional spread of *C. burnetii,* but with different capacities. On the one hand, airborne transmission had the ability to introduce *C. burnetii* in a large number of herds, but the generated outbreaks were generally predicted to be ephemeral and small. On the other hand, animal trade was predicted to result in only 8% of new infections; however, purchasing an infectious cow could instigate comparatively larger outbreaks. The differences in the impact of each transmission route on the intra-herd infection dynamics arose from the intrinsic nature of these transmission routes in spreading *C. burnetii*.

Regardless the route, the first generated local infection was always a cow with health status *I*^−^ as shown in Figure 2. Such a seronegative shedding cow is a transient shedder, which can become susceptible again. Therefore, in herds in which cows were infected by airborne transmission, infection might go extinct if the transient first local infection does not shed enough to generate subsequent infections, which are essential for infection persistence. In herds introducing infectious cows by trade, the animal purchased could be either a transient shedder (*I*^−^) or a permanent shedder (*I*^+^ or *I*^*milk pers*^). Hence, after the generation of the first local infection, there are at least two shedding cows in herds purchasing infectious animals, leading to potentially higher bacterial contamination and increasing the probability of intra-herd infection persistence.

Our results were consistent with those of a previous study [18] based on a statistical regression model. This study indicated that airborne transmission and cattle trade are both risk factors for the dairy cattle herds in the Finistère department. It also attributed higher proportion of infections to airborne transmission than to animal movements in areas with high cattle density. A cluster analysis performed in the same study for the 2012 seroprevalence in dairy herds showed a high-risk cluster in the north-western corner of the Finistère department [18]. According to our findings, predicted probabilities of herd infection in 2013 exhibit two high-risk clusters in the same area, known to have a high density in cattle.

The contribution of animal trade to livestock pathogen transmission is known to vary considerably. For Q fever, cattle trade seems to explain quite a low proportion of incidence compared to airborne transmission, at least in areas with a high cattle density and high prevalence. However, trade is known to play an important role in the regional spread of other pathogens, such as the foot-and-mouth disease virus (FMDV) and the bovine viral diarrhoea virus [30, 31]. For bovine tuberculosis—as here for Q fever, trade is correlated to a low number of infections [32] compared to other transmission routes.

While these studies focused on the regional contribution of transmission pathways, we also highlighted differences in intra-herd infection dynamics depending on these pathways. The simulated differences in the intensity of intra-herd outbreaks experienced by herds acquiring infection by cattle trade and by airborne transmission, and the capacities of these routes to affect infection-free herds provide valuable insights for risk assessment. Even if cattle trade does not seem to contribute largely to new infections, preventing the purchase of infected animals is still a relevant measure to limit infection spread at the intra-herd scale. In addition, new infections could be caused by airborne transmission from herds infected by import of infected livestock.

From the model perspective, it is the first time, to our knowledge, that a Gaussian dispersion model for infectious particles is coupled with an intra-herd infection dynamics model to describe the spread of an enzootic livestock disease. Gaussian dispersion models have been employed previously in the description of the spread of livestock-related viruses such as FMDV and the avian influenza virus [33, 34]. In another study, a dispersion model was used to quantify the possible risk of Q fever in humans from nearby infected sheep farms [35].

One of the main advantages of using mechanistic models is that they allow identifying the source of infection, and subsequently help implementing targeted intervention measures [36]. The mechanistic model presented here allowed identifying the cause of infection of susceptible herds based on the dominant contributory route, at the time of generation of the first local infection. Moreover, according to our investigations, the combined effect of the two processes (airborne transmission and animal trade) at a regional scale is additive and not synergistic, at least over a short period of time.

Performance measures of the model at the neighbourhood level can be interpreted as the model’s ability to predict an observed herd infection within a given area. The increase in the AUC for the comparisons done at different neighbourhood radii also indicates the model’s ability to capture the spatial nature of the dispersion. Assuming that the neighbourhood range and the accuracy of the model depend on the herd density and the clustering of the infection in the region studied, selection of a neighbourhood range becomes case-specific. The ROC analysis performed for different neighbourhoods is an effort to increase the sensitivity of the model without altering its specificity, with more weight given to the capacity of the model to identify positive herds. The sensitivity of the model hence increases with the decreasing spatial granularity.

Irrespective of the benefits, mechanistic models are generally difficult to fit to data. Spatio-temporal outcome of FMDV models, when tested against the 2001 outbreak data, have shown about 10–15% accuracy [37]. In the current Q fever model, higher accuracy of the model is probably due to the high prevalence and the enzootic nature of the infection in the study region. Models are generally used to simulate the overall spread of pathogens to produce expected epidemic curve, and are often difficult to be judged for their relevance, especially in the absence of detailed and accurate data. Since many models are increasingly depicting the spatial spread of pathogens in livestock in enzootic regions, more refined evaluation of their ability to produce spatial patterns in agreement with field observation needs to be performed. Analysis based on ROC spatial analysis, like the one used here, can be useful in understanding the complex spatial behaviours of such models.

Although we cannot deny the possible existence of interactions between the tested parameters with potential impact on model outputs, the univariate sensitivity analysis performed supports the relative robustness of model predictions at the elementary level. The main output of the model concerning the relative contributions of the transmission routes in the regional spread of *C. burnetii* showed moderate perturbations to parameter variations, especially when the plume was generated at rates high enough (allowing airborne transmission) compared to death rate of bacteria (parameter *κ* related to the ratio between these two rates). To reduce the uncertainty on these parameters and hence on their effect on the infection dynamics, more data collection is essential to estimate the bacterial quantities generally found in and leaving farm buildings. The possible effects of super shedders were indirectly assessed using sensitivity analysis of the model to *Q1*, which is the probability distribution of the shedding levels for all the *I*^−^ and for the *I*^+^ shedding in mucus/faeces after four weeks post-calving. Two of the probability distributions tested (described in Table 4) assumed proportions of high shedders of 0.25 (distribution IV) and 0.5 (distribution III), whereas the reference scenario assumed no high shedders in these classes. It seems that in scenarios corresponding to distributions III and IV for *Q1* the contribution of trade was diminished, but this needs to be confirmed in further refined analysis.

The model is expected to underestimate the spread of *C. burnetii* as we ignore beef herds in the study region, which can transmit *C. burnetii* to dairy cattle herds by airborne transmission, and also as we consider cattle trade within the concerned department only. Indeed, according to the analysis of a larger database over the period 2005–2009, 22% of all the concerned transactions of cows involving dairy herds located in the Finistère department corresponded to purchases from outside the department (personal communication: B. L. Dutta). However, no epidemiological information was available for these herds. Similarly, the impact of small ruminant flocks was also neglected as very few such flocks are present in the region. Accuracy of the model could be further improved if epidemiological data about beef herds and other livestock flocks in and around the region were available.

The time-varying nature of the network describing cattle trade, in particular the large variability in the trade relationships between herds from one year to the next (as described by Dutta et al. [38]), suggests that the transmission route due to trade could have a lager impact on the regional dynamics over a longer duration. Indeed, new susceptible target herds could be linked to the network of herds by enlarging the time window of the study. Similarly, the capacity of airborne transmission of the bacteria is relatively unhindered and all herds get exposed in a very densely populated region without any geographical barriers such as in the Finistère. Hence, the regional spread and corresponding control strategies predominantly depend on the prevalence of infection, characteristics of the cattle trade network, and cattle density. On the backdrop of these, the model presented here can become a useful tool to control the regional spread of *C. burnetii* by assessing the impact of relevant interventions such as vaccination of cows [39] and testing of cows for the presence of *C. burnetii* before trading.

## Additional files

10.1186/s13567-016-0330-4 **Model equations and incorporation of data in the model.** Additional file 1 contains following sections: (1) The intra-herd model: Eqs. (2) Airborne transmission of infectious particles in herd neighbourhood and incorporation of meteorological data [40, 41] . (3) Estimation of the optimum cut-off values for probability of infection (PI) for incident herds
10.1186/s13567-016-0330-4 **Performance of the model concerning the choice of PI cut-off optimal values at herd and neighbourhood levels.** Selection of optimum PI cut-off is based on three criteria in ROC analysis.
10.1186/s13567-016-0330-4 **Incidence predicted at cut-offs of 0.11 and 0.61 (optimum PI values for herd level analysis) and at 0.21, 0.22 and, 0.15 (optimum PI values for a neighbourhood of 3** **km).** Maps of the Finistère department (France) show herds predicted positive in 2013 at different possible PI cut-off values.

## Abbreviations

AUC: area under the curve
BTM: bulk tank milk
ELISA: enzyme-linked immunosorbent assay
FMD: foot and mouth disease
ROC: receiver operating characteristic
