# Supplementary material for: Spread of Coxiella burnetii between dairy cattle herds in an enzootic region: modelling contributions of airborne transmission and trade
Source: Vet Res. 2016 Apr 5;47:48. doi: 10.1186/s13567-016-0330-4 (PMC4822316; doi:10.1186/s13567-016-0330-4)
Supplement: Supplementary file 1 — 10.1186/s13567-016-0330-4 Model equations and incorporation of data in the model. Additional file 1 contains following sections: (1) The intra-herd model: Eqs. (2) Airborne transmission of infectious particles in herd neighbourhood and incorporation of meteorological data [40, 41] . (3) Estimation of the optimum cut-off values for probability of infection (PI) for incident herds [file 13567_2016_330_MOESM1_ESM.docx]

1. The intra-herd model: equations

The model for dynamics of *Coxiella burnetii* in a dairy cattle herd used in this study is a variant of the model introduced by Courcoul et al. in 2011 [10].

Here we present the Equations 1-6, which describe the updating (between time steps (*t-1*) and *t*) of variables corresponding to health states of cows in a herd *i*:

$S_{i}\left( t \right)= S_{i}\left( t-1 \right)-NI_{i}^{-}\left( t \right)+NS_{i}\left( t \right)+\Omega[S_{i}]\left( t-1 \right)$ (1)

$I_{i}^{-}\left( t \right)=I_{i}^{-}\left( t-1 \right)+NI_{i}^{-}\left( t \right)-NS_{i}\left( t \right)-NI_{i}^{+}\left( t \right)-NI_{i}^{+milk pers}\left( t \right)+\Omega{[I}_{i}^{-}]\left( t-1 \right)$ (2)

$I_{i}^{+}\left( t \right)= I_{i}^{+}\left( t-1 \right)+NI_{i}^{+}\left( t \right)-NI^{+}C_{i}^{+}\left( t \right)+NC^{+}I_{i}^{+}\left( t \right)+NC^{-}I_{i}^{+}\left( t \right)+\Omega{[I}_{i}^{+}]\left( t-1 \right)$ (3)

$I_{i}^{+ milk pers}\left( t \right)=I_{i}^{+milk pers}\left( t-1 \right)+NI_{i}^{+milk pers}\left( t \right)-NI^{+milk pers}C_{i}^{+}\left( t \right)+\Omega[I_{i}^{+milk pers}]\left( t-1 \right)$ (4)

$C_{i}^{+}\left( t \right)= C_{i}^{+}\left( t-1 \right)+NI^{+}C_{i}^{+}\left( t \right)+NI^{+milk pers}C_{i}^{+}\left( t \right)-NC^{+}I_{i}^{+}\left( t \right)-NC^{+}C_{i}^{-}\left( t \right)+\Omega{[C}_{i}^{+}]\left( t-1 \right)$ (5)

$C_{i}^{-}\left( t \right)= C_{i}^{-}\left( t-1 \right)+NC^{+}C_{i}^{-}\left( t \right)-NC^{-}I_{i}^{+}\left( t \right)+\Omega{[C}_{i}^{-}]\left( t-1 \right)$ (6)

Based on Equations 1-6, it is possible to define two main outputs of the model at the herd level as:

${Seroprevalence}_{i}\left( t \right)=I_{i}^{+}\left( t \right)+I_{i}^{+ milk pers}\left( t \right)+C_{i}^{+}\left( t \right)$ (7)

${Shedders}_{i}\left( t \right)=I_{i}^{-}\left( t \right)+I_{i}^{+}\left( t \right)+I_{i}^{+ milk pers}\left( t \right)$ (8)

Equations 9-16 here below define ingoing and outgoing flows in Equations 1-6, with parameters defined in Table 2 (main text):

$NI_{i}^{-}\left( t \right)\sim Bin (S_{i}\left( t-1 \right), p_{i}(t))$*, where* $p_{i}\left( t \right)= 1-e^{-(E_{i}(t-1))}$ (9)

$(NS_{i}\left( t \right),NI_{i}^{+}, NI_{i}^{+ milk pers})\sim Multinomial (I_{i}^{-}\left( t-1 \right), (\frac{m}{m+q}, \frac{qpIp}{m+q}, \frac{q(1-pIP)}{m+q}))$ (10)

$NI^{+}C_{i}^{+}\left( t \right)\sim Bin (I_{i}^{+}\left( t-1 \right),r_{1} )$ (11)

$NI^{+milk pers}C_{i}^{+}\left( t \right)\sim Bin (I_{i}^{+milk pers}\left( t-1 \right),r_{2} )$ (12)

$(NC^{+}C_{i}^{-}\left( t \right),NC^{+}I_{i}^{+}\left( t \right))\sim Multinomial (C_{i}^{+}\left( t-1 \right), (\frac{\tau}{\tau+s}, \frac{s}{\tau+s}))$ (13)

$NI_{i}^{-}\left( t \right)\sim Bin (S_{i}\left( t-1 \right), p_{i}(t))$ (14)

$NC^{-}I_{i}^{+}\left( t \right)\sim Bin (C_{i}^{-}\left( t-1 \right), p_{i}(t))$ (15)

$\Omega\left[ X \right]_{i}\left( t \right)=\sum_{j=1}^{Nb} Multinomial (n_{ji}\left( t \right),P_{X,ji}\left( t \right))-\sum_{j=1}^{Nb} Multinomial (n_{ij}\left( t \right), P_{X,ij}\left( t \right))$ (16)

where*,* $, X\in\{S,I^{-}, I^{+}, I^{milk pers}, C^{+}, C^{-}\}$ and

$P_{X,ij}(t)= \left[ \frac{S_{i,l}(t)}{N_{i,l}\left( t \right)} , \frac{I_{i,l}^{-}(t)}{N_{i,l}\left( t \right)} ,\frac{I_{i,l}^{+}(t)}{N_{i,l}\left( t \right)}, \frac{I_{i,l}^{milk pers}(t)}{N_{i,l}\left( t \right)}, \frac{C_{i,l}^{+}(t)}{N_{i,l}\left( t \right)} , \frac{C_{i,l}^{-}(t)}{N_{i,l}\left( t \right)} \right]$ is the probability of selling a cow in the lactation year *l* and with specific health status to herd *j.* The lactation year (*l)*, $the$number of purchases made by herd *I* from *j (*$n_{ji})$, and the number of cows sold to herd *j* by herd *i* ($n_{ij}$) were based on the data.

The dynamics of bacterial load in the environment is given by the following equation (17, identical to Equation 3 in the main text):

$E_{i}\left( t \right)=E_{i}\left( t-1 \right)\left( 1-\mu\right)+E_{i,internal}(t-1)+E_{i,external}(t-1)+\sum_{j} E_{i,j,dep}(t-1)$ $E_{i}(t)=E_{i}(t-1)\left( 1-\mu\right)+\mathrm{Bact}_{i, Local}(t-1)+\mathrm{Bact}_{i,Foreign}(t-1)+\sum_{j} \mathrm{Bact}_{Dep, i,j}(t-1)$ (17)

The overall dynamics of the environmental bacterial load is governed by animals shedding through different routes at different stages of their reproductive cycle and at different levels of shedding. According to [10], for herd *i* this hence can be summarized in the following equation:

${Bact}_{i,x,origin}(t)= {\sum_{k, l} \left( \rho^{k}{Qty}_{l}\sum_{i, j} n_{t, xwkl} \right)}_{origin}$, (18)

where, *origin* $\in\left\{ internal, external \right\}; x\in\left\{ I^{-}, I^{+}, I^{milk pers} \right\}$are the different health states of cows which can shed the bacteria, $w\in\left\{ \leq4 weeks post calving , >4 weeks post calving \right\}$ is the state of reproductive cycle of the cow, $k\in\left\{ milk,mucus/feaces \right\}$, is the route by which the quantities *Qty_l_* of bacteria are shed, $l\in\left\{ low, medium, high \right\}$ is the level of bacterial shedding; $n_{t,xwkl}\sim Multinomial \left( N_{t,xwk}, {Qc}_{(x,w,k)} \right)$ with $N_{t,xwk}$ the number of animals in corresponding health state at time *t* and ${Qc}_{(x,w,k)}$ the probability distributions governing shedding levels. The remaining parameters are defined in Tables 2 and 3 of the main text.

1. **Airborne dispersion of infectious particles in herd neighbourhood and incorporation of meteorological data**

The wind speed and direction data from the European Centre for Medium Range Weather Forecasts database were procured for the entire Finistère department (Western France). Data consisted of daily values of northward wind component and eastward wind component. Using this data, the wind speed was estimated as $windspeed= \sqrt{{northward wind component}^{2}+{eastward wind component}^{2}}$, and direction of the wind flow was estimated through its angle ɸ with the original x-axis, where $ɸ= \tan^{-1} \frac{northward wind component}{eastward wind component}$. Weekly averages of wind direction and speed (a unique value for the whole area considered, here the Finistère department) were used in the Gaussian dispersion model. Adjustment of the frame of the receiving *j* and source *i* herd coordinates according to the direction of wind flow was done based on the distance between the two herds (distance_ij_), the angle between the line linking the two herds and the x-axis (angle_ij_) and angle ɸ, as described in Stockie [27]: $x_{adjusted}={distance}_{ij}* \cos\left( {angle}_{ij}-ɸ \right)$ and $y_{adjusted}={distance}_{ij}* \sin\left( {angle}_{ij}-ɸ \right)$.

The source for generation of the plume leaving the source herd *i* was the bacteria being lost from the environment compartment of this herd (rate of bacterial removal from the environment due to various mechanisms estimated for the intra-herd dynamics by Courcoul et al. [23]). Here, we assume that a part of bacteria shed by cows through different routes form dust particles, which remain infectious and become source for generation of plume. This plume then transported by the wind to another herd. Indeed, the smaller droplets generated by sneezing, coughing, splashing and other activities remain suspended in the air and dry fast enough to produce smaller particles called droplet nuclei, which can remain suspended in the air for long duration and can be transported along with the wind to distant places, unlike larger particles. Hence, the inherent capacity of airborne transmission of any infectious agent depends on production of appropriate range of droplet particle sizes with viable pathogens [40]. Multiple studies have suggested a higher risk of airborne transmission of Q fever within the radius of 5 km from the source in moderate environmental conditions [41, 35]. Hence, we restricted our dispersion model to a radius of 5 km from the source herd.

1. **Estimation of the optimum cut-off value for probability of infection (PI) for incident herds**

The cut-off PI was used to classify the herds into two categories: herds with a PI larger than the cut-off were considered positive (infected), the others were negative (uninfected). This categorisation concerns the simulated herd status at one year after the onset of pathogen spread into the metapopulation. The optimum cut-off is chosen based on comparison of simulation to data concerning herd status at time zero (of the follow-up) and at one year. The optimum cut-off value is chosen based on the epidemiological situation of the case concerned, such as prevalence in the population and consequences of false positive and false negative results. In the literature, prevalence dependent (Sensitivity (*Se*), Specificity (*Sp*), Youden index (*J*), odds ratio etc) and independent criteria (Efficiency, kappa) are both used to come up to a decision [29]. Here, three criteria were used to determine the optimal cut-off value: $Se = Sp$, maximum Youden index (J_max_, where $J=Se+Sp-1$) and maximum accuracy (Acc_max_, where Acc is equal to the proportion of true negative and true positive into the population). Values of these criteria along with PI cut-off values are provided in Additional file 2 for both herd level and neighbourhood (3 km) based on simulations (description of the simulation settings in the main text).

Predicted incident herds for different values of cut-off at one year after the onset of pathogen spread into the metapopulation, specifying the contamination route, are provided in Additional file 3.
