# Supplementary material for: Spread of Coxiella burnetii between dairy cattle herds in an enzootic region: modelling contributions of airborne transmission and trade
Source: Vet Res. 2016 Apr 5;47:48. doi: 10.1186/s13567-016-0330-4 (PMC4822316; doi:10.1186/s13567-016-0330-4)
Supplement: Supplementary file 2 — 10.1186/s13567-016-0330-4 Performance of the model concerning the choice of PI cut-off optimal values at herd and neighbourhood levels. Selection of optimum PI cut-off is based on three criteria in ROC analysis. [file 13567_2016_330_MOESM2_ESM.docx]

Additional file 2 Performance of the model concerning the choice of PI cut-off optimal values at herd and neighbourhood levels. (Values in bold are PI values at which criteria were fulfilled).

|  | **Herd Level** | | | **Neighbourhood (3 km)** | | |
| --- | --- | --- | --- | --- | --- | --- |
| **Criteria** | Se≈Sp | Acc_max_ | J_max_ | Se≈Sp | Acc_max_ | J_max_ |
| PI cut-off | **0.11** | **0.61** | **0.11** | **0.21** | **0.25** | **0.15** |
| Sensitivity | **0.58** | 0.10 | 0.58 | **0.75** | 0.71 | 0.86 |
| Specificity | **0.58** | 0.95 | 0.58 | **0.75** | 0.80 | 0.66 |
| Accuracy | 0.58 | **0.64** | 0.58 | 0.75 | **0.76** | 0.73 |
| Youden index (J) | 0.15 | 0.06 | **0.15** | 0.51 | 0.51 | **0.53** |
| Incidence | 419 | 58 | 419 | 259 | 219 | 346 |
| % airborne transmission | 86 | 57 | 86 | 78 | 75 | 83 |
