# Supplementary figures and images for: Spread of Coxiella burnetii between dairy cattle herds in an enzootic region: modelling contributions of airborne transmission and trade
Source: Vet Res. 2016 Apr 5;47:48. doi: 10.1186/s13567-016-0330-4 (PMC4822316; doi:10.1186/s13567-016-0330-4)

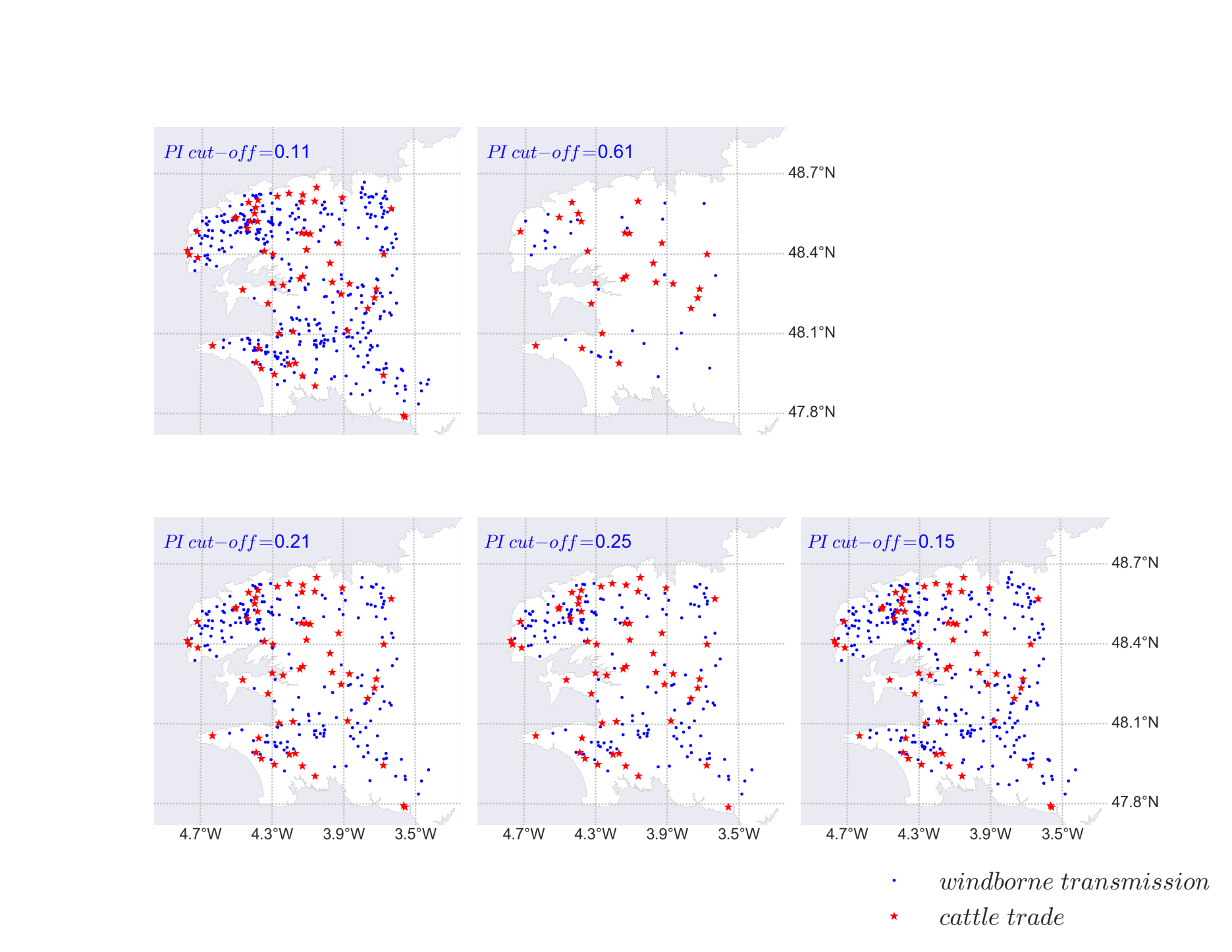

Supplement: Supplementary file 3 — 10.1186/s13567-016-0330-4 Incidence predicted at cut-offs of 0.11 and 0.61 (optimum PI values for herd level analysis) and at 0.21, 0.22 and, 0.15 (optimum PI values for a neighbourhood of 3 km). Maps of the Finistère department (France) show herds predicted positive in 2013 at different possible PI cut-off values. [file 13567_2016_330_MOESM3_ESM.docx]
